# Parental Attributions of Control for Child Behaviour and Their Relation to Discipline Practices in Parents of Children with and Without Developmental Delays

**DOI:** 10.1007/s10826-017-0676-x

**Published:** 2017-03-08

**Authors:** Myrthe Jacobs, Lisa Marks Woolfson, Simon C. Hunter

**Affiliations:** 1 School of Psychological Sciences and Health, University of Strathclyde, 6th floor, Graham Hills Building, 40 George Street, Glasgow, G1 1QE United Kingdom; 2 Graduate School of Education, The University of Western Australia, Perth, Australia; 3 Centre for Health Policy, University of Strathclyde, Glasgow, United Kingdom

**Keywords:** Developmental delay, Parenting, Child behavior, Causal attributions, Control

## Abstract

Children with developmental delays (DD) are at risk for developing behavior problems. Research suggests that parents’ causal attributions for child behavior are related to parenting. This study investigated this association in parents of children with DD compared to parents of typically developing (TD) children. It specifically focused on attributions of child control by separating these from attributions of responsibility, blame and intent, and from attributions of parent control and responsibility. Fifty-one parents of children with DD and 69 parents of TD children completed two questionnaires. The Written Analogue Questionnaire measured causal attributions. The Parenting Scale measured dysfunctional discipline practices. Parents of children with DD viewed the child’s role in problematic behavior more positively while also viewing misbehavior as more fixed than parents of TD children. Parents of TD children who viewed their child as more in control over misbehavior used less dysfunctional discipline, but this association was not found for parents of children with DD. The results advance understanding of how parents perceive behavior problems in children with DD and the important role these perceptions play in parental behavior management strategies. More importantly, these perceptions relate to discipline practices differently for parents of children with DD compared to parents of TD children, highlighting that parent interventions should be adapted to the specific needs of parents of children with DD.

## Introduction

Children with developmental delays (DD) experience higher rates of behavior problems than children without DD, including hyperactivity, emotional problems and conduct problems (Totsika et al. 2011). Noncompliance, although a normal part of child development, is among the most prevalent and is a considerable problem for parents of children with DD (Mitchell and Hauser-Cram 2009; Taanila et al. 2003; Walker 1993). Child behavior and parent behavior affect each other, but parent behavior is also influenced by parental beliefs (Sameroff and Fiese 2000). One way of examining parental beliefs on child behavior is through a causal attributional model (Miller 1995).

Attributional theory focuses on how people think about causes of behavior and how this relates to their reactions towards this behavior (Heider 1944). Weiner developed the theory by formulating and testing dimensions to which causes for behavior can be ascribed, and by explaining how these attributions cause emotions and behaviors in response to perceived behavior (Weiner 1979, 1980, 1985).

Judgements of control and responsibility are related but distinct (Weiner 1995). While attributions of control represent characteristics of causes, responsibility indicates a judgement about a person. Several factors are taken into account when a responsibility judgement is made. The cause of the behavior must be seen as controllable, and the more the behavior is perceived as intentional, the more they will be judged to be responsible (Heider 1958; Shaver 1985; Weiner 1995). Additionally, the person must be viewed as having awareness of the consequences and the moral implications of the behavior (Heider 1958; Shaver 1985). For this reason, younger children and children with DD might not be held responsible for actions they intended and are in control of. Blame follows from a judgement of responsibility when the consequence of the behavior is significant enough and when justifications or excuses presented by the person are not accepted (Shaver 1985; Weiner 1995). The main difference between responsibility and blame is that while responsibility is neutral in affect, blame suggests emotional negativity and is seen as a combination of responsibility and anger (Weiner 1995).

Control-related causal attributions for child behavior have been found to relate to parenting. In general, parents of typically developing (TD) children attribute low levels of control to their child for problematic behavior (Cote and Azar 1997; Dix et al. 1986; Gretarsson and Gelfand 1988; Mills and Rubin 1990). A view of the child as low in control, intent, blame, and responsibility for misbehavior in comparison to a view of the child as high in these attributions, is associated with the tendency of parents of TD children to experience less negative affect, to express less anger and disapproval, and to use less physical aggression (Dix et al. 1986, 1989; Slep and O’Leary 1998; Snarr et al. 2009).

In comparison to parents of TD children, parents of children with DD can additionally attribute behavior to the DD itself (Armstrong and Dagnan 2011; Drysdale et al. 2009; Jacobs et al. 2015; Keenan et al. 2007; Whittingham et al. 2008). Parents may view problematic behavior as an inevitable part of the child’s condition, leading parents to accept problematic behaviors from the child (Woolfson 2004). Viewing the child’s condition as a cause for difficult behavior could therefore be disadvantageous for motivation towards behavior change (Peters et al. 2005). However, none of the above studies have included a comparison of attributions between parents of TD children and children with DD.

Relations between control-related causal attributions and parenting strategies have been reported for parents of children with DD. Parents rated the usability of behavior management strategies more highly when they viewed child behavior as caused by factors that were less controllable by the child (Whittingham et al. 2006). Attributions of child responsibility related positively to parents’ negative emotional reactions, aggressive behavior, and likelihood to punish a child (Armstrong and Dagnan 2011; Chavira et al. 2000).

Some of these study samples were, however, very specific. Drysdale et al.’s study (2009) focused on white British mothers of children with DD who experienced self-injurious behavior. Keenan et al. (2007) recruited parents of children with DD who experienced sleep problems. Studies by Whittingham et al. (2006, 2008) were with parents of children with ASD and only mothers from Latin-American descent with low socio-economic backgrounds participated in Chavira et al.’s study (2000). This limits the generalizability of findings to wider DD groups.

Based on these studies, it appears that for both parents of TD children and children with DD, viewing the child as having low levels of control and low levels of responsibility would be beneficial for parental emotional and behavioral reactions. However, it has also been suggested that attributing low levels of control to the child would lead to parents feeling low levels of responsibility for managing the child’s behavior (Gretarsson and Gelfand 1988). The attribution of both high and low levels of control to the child has been theorized to lead to lower levels of participation by parents in treatment processes (Morrissey-Kane and Prinz 1999; Smith et al. 2000), and to be disadvantageous to motivation for changing child behavior (Hoza et al. 2006; Mah and Johnston 2008). Woolfson’s “parenting paradox” (2005) argues that attributing either low or high control to the child is not desirable; rather, parents need to attribute moderate levels of control to view their child as capable of learning new behaviors.

In addition to beliefs on the child’s role in behavior, parents have beliefs on their own levels of control over child behavior and these have been found to relate to their emotional and behavioral reactions. Parents of TD children who hold themselves responsible for problematic child behavior experience more negative affect, and are more likely to overreact and to use physical aggression (Snarr et al. 2009). Those who attribute less control to themselves hold lower expectations for the effectiveness of parenting strategies (Baden and Howe 1992; Sobol et al. 1989). In particular, low perceived control, a combination of low parental control and high child control, relates to harsh and coercive parenting (Bugental et al. 1989; Bugental and Happaney 2004). These relations between parental attributions of control and responsibility to themselves for child behavior have not been assessed among parents of children with DD.

Another limitation of the research carried out on control-related causal attributions is the variation in forms of assessment that have been employed across studies. For example, while asking about control can activate other related beliefs, such as responsibility, intent and blame, these terms have sometimes been used interchangeably (Fincham and Roberts 1985; Mantler et al. 2003), or have been examined using summary scales composed of items measuring, control, intent, or blame (Chavira et al. 2000; Slep and O’Leary 1998; Snarr et al. 2009). To understand the differences between these control-related beliefs and their relationships with parenting, attributions of control, responsibility, intent and blame need to be clearly separated.

This study therefore aimed to compare parents of children with DD and TD children on causal attributions of child control, responsibility, intent and blame, and parent control, responsibility and anger, and on how problematic they view behavior. It also aimed to examine the relations of these variables with parenting practices in parents of children with DD and TD children. As parents of children with DD have an additional cause to attribute behavior to in comparison to parents of TD children, it was hypothesized that their control-related causal attributions directed to the child and the parent would differ from each other. A comparison of attributions between parents of TD children and children with DD has not been made before, and therefore the direction of effects was not specified. Based on the results of previous research, it was hypothesized that attributions of higher levels of control, responsibility, blame and intent attributed to the child, and higher level of responsibility and lower levels of control attributed to the parent, would be related to the use of less effective discipline practices.

## Method

### Participants

Recruitment was through schools, parent websites, and children’s play centers. The only inclusion criterion was being a parent or carer of a child with DD or TD child aged 6–12 years. This resulted in a sample of 51 parents of children with DD and 69 parents of TD children (100 mothers, eight fathers, 12 carers). Parents of children in the DD-group reported the following diagnoses: autism (*n* = 17), Down syndrome (*n* = 12), cerebral palsy (*n* = 4), Angelman syndrome (*n* = 1), and chromosomal disorders (*n* = 4). A further 13 DD-group parents reported their children to have a general developmental delay without an additional diagnosis.

Parents were asked to estimate their child’s developmental level by answering the following question “How would you estimate your child’s development in comparison to any typical child his/her age” by checking one of the five following options: (1) severe delay (2) moderate delay (3) mild delay (4) typical development (5) advanced for age. All parents in the DD-group estimated their child’s development as moderately or severely delayed in comparison to a TD child. Children of TD-group parents did not have any such diagnoses or epilepsy, ADHD, autism spectrum disorder or a specific learning problem. These parents estimated their child’s development as typical or advanced compared to children the same age.

Table 1 shows that groups differed significantly on gender and income, as in previous studies (Leonard and Wen 2002; Maul and Singer 2009). In comparison to the TD-group, DD-group children were more likely to be male, and DD-group parents were more likely to have lower incomes.

**Table 1:** Sample demographic information

				Test statistics	
		DD	TD	DD vs. TD	
Child age *M* (SD; *range*)		8.7 (1.62; 5.5–12.5)	8.4 (1.66; 5.9–11.7)	*t*(116) = 0.98	
Child gender *n* (%)	Boy	39 (76.5)	36 (52.2)	χ^2^(1) = 8.30^**^	
	Girl	11 (21.6)	33 (47.8)		
	Missing	1 (2.0)	0 (0.0)		
School *n* (%)	Mainstream	7 (13.7)	69 (100)	N/A	
	Specialist	36 (70.6)	0 (0.0)		
	Specialist unit	6 (11.8)	0 (0.0)		
	Both spec. and main.	2 (3.9)	0 (0.0)		
Relation to child *n* (%)	Mother	42 (82.4)	58 (84.1)	χ^2^(2) = 0.06	
	Father	6 (11.8)	2 (2.9)		
	Carer	3 (5.9)	9 (13.0)		
Ethnic background *n* (%)	White	45 (88.2)	67 (97.1)	N/A	
	Asian/Asian British	4 (7.8)	0 (0.0)		
	Black(British)/African/Caribbean	1 (2.0)	0 (0.0)		
	Mixed/multiple groups	1 (2.0)	2 (2.9)		
Level of education *n* (%)	Primary/secondary	11 (21.6)	10 (14.5)	χ^2^(3) = 4.16	
	Highers/college	7 (13.7)	16 (23.2)		
	Degree/diploma	18 (35.3)	22 (31.9)		
	Postgraduate	8 (15.7)	20 (29.0)		
	Missing	7 (13.7)	1 (1.4)		
Marital status *n* (%)	Never married	5 (9.8)	6 (8.7)	χ^2^(1) = 0.01	
	Cohabiting	6 (11.8)	6 (8.7)		
	Currently married	32 (62.7)	46 (66.7)		
	Separated	2 (3.9)	4 (5.8)		
	Divorced	4 (7.8)	3 (4.3)		
	Widowed	2 (3.9)	1 (1.4)		
	Missing	0 (0.0)	3 (4.3)		
Occupation *n* (%)	Paid employment	25 (49.0)	43 (62.3)	χ^2^(1) = 3.33	
	Self employed	3 (5.9)	4 (5.8)		
	Non-paid, volunteer	1 (2.0)	1 (1.4)		
	Student	2 (3.9)	1 (1.4)		
	House maker	14 (27.5)	15 (21.7)		
	Unemployed	1 (2.0)	2 (2.8)		
	Other	5 (9.8)	0 (0.0)		
	Missing	0 (0.0)	3 (4.3)		
Income in GBP *n* (%)	up to £15,000	19 (37.3)	14 (20.3)	χ^2^(3) = 7.74^*^	
	up to £30,000	14 (27.5)	18 (26.1)		
	up to £45,000	11 (21.6)	15 (21.7)		
	£45,000 and above	4 (7.8)	17 (24.6)		
	Missing	3 (5.9)	5 (7.2)		

^*^ *p* < .05; ^**^ *p* < .01.

### Procedure

Ethical permission was gained from the appropriate ethics committee at the administering institution. Parents received an information sheet, consent form and the questionnaire pack in their child’s school bag and returned completed questionnaires to their child’s school where they were collected. Contact details of the researcher were provided. For the online questionnaire, parents found the link on forums or in newsletters and could complete the questionnaire online. In addition, play centers were approached and the researcher visited these to ask parents individually to complete questionnaires. Parents were able to skip any questions they preferred not to answer. Informed consent was gained from all parents. Completion of the questionnaires took 30 to 45 min.

### Measures

#### Written analogue questionnaire

The Written Analogue Questionnaire (WAQ) assesses parents’ attributions for child behavior using vignettes (e.g. Johnston and Freeman 1997; Johnston et al. 2009). Vignettes can be adapted for different populations (Johnston and Freeman 1997). For the present study, three vignettes were selected that have been adapted from the WAQ for use in research with parents of children with intellectual disability (Jacobs et al. 2015). The three vignettes are displayed in Table 2.

**Table 2:** WAQ vignettes and items

Vignettes used from the WAQ as adapted by Jacobs et al. (2015)	
Vignette 1	Your child is looking for a certain toy he wants to play with while you are busy talking on the telephone. When he can’t find it, he tries to get your attention and keeps interrupting you to indicate that he wants you to help him find the toy.
Vignette 2	You and your child are in the lounge. You are planning a family outing that day and together you are waiting for the weather forecast on the TV. Just as the weather comes on, your child begins to make a noise with a toy that he is playing with.
Vignette 3	Your child is getting ready for school. You notice that his hair is not yet brushed. You remind him that his hair needs to be brushed before going to school but he refuses and does not cooperate.

Questionnaire item		Anchor
Problem	How much of a problem did you feel the behavior was?	not at all—very much
Child control	To what extent was your child’s behavior caused by something within his or her control?	completely within his or her control—not at all within his or her control
Child responsibility	Is your child responsible for the way in which s/he behaved?	not at all responsible—very much responsible
Blame	Is your child to blame for what s/he did?	not at all to blame—very much to blame
Intent	Did your child behave this way on purpose?	not at all—very much
Parent control	To what extent was your child’s behavior caused by something within your control?	completely within my control—not at all within my control
Parent responsibility	To what extent were you responsible for your child’s behavior?	not at all responsible—very much responsible
Anger	How angry did you feel when you saw your child act this way?	not at all—extremely

After reading each vignette, parents rated individual items measuring *child control*, the extent to which the parent felt the behavior was a problem, and *parent responsibility* for the behavior. An item for *parent control* was added (adapted from Walker 1985). Three items measuring *child responsibility*, *intent*, and *blame* (adapted from Chavira et al. 2000) were added and parents rated feelings of anger (see Table 2). All items had Likert-scales from 1 to 10. Attribution scales were composed by calculating average scores across the three vignettes. Cronbach’s *α* for all scales ranged from .64–.87 for the present sample, indicating acceptable reliability.

#### Parenting scale

The Parenting Scale (PS) (Arnold et al. 1993) is a 30-item self-report questionnaire assessing dysfunctional discipline practices for general problematic child behavior. Each item consists of a 7-point scale which proposes a discipline ‘mistake’ on one anchor and its more effective equivalent on the other anchor. It has a Total score and three sub-scales. *Laxness* measures permissive discipline, e.g. ‘When I say my child can’t do something, I let my child do it anyway—I stick to what I said’. *Overreactivity* measures anger and irritability, e.g. ‘I get so frustrated or angry that my child can see I’m upset—I handle it without getting upset’. Finally, *verbosity* measures lengthy ineffective verbal responses, e.g. ‘I give my child a long lecture—I keep my talks short and to the point’. Cronbach’s *α* for the Total scale in the present sample was .85, indicating good reliability. The Parenting Scale Total score reflects a global index of dysfunctional parenting and higher scores indicate parenting that is more dysfunctional (Arnold et al. 1993).

#### Data analyses

*Perceived control* scores (Bugental et al. 1989; Bugental and Happaney 2004) were derived by rescoring and then subtracting *child control* from *parent control* scores. This resulted in the *perceived control* construct where a score near zero indicated a balance between child and parent control, a positive score indicated high *parent control* with low *child control*, and a negative score indicated low *parent control* with high *child control*.

## Results

In order to test for group differences on causal attributions a set of 10 analyses were conducted using MPlus 7.31 (Muthén and Muthén 2015). In each analysis, a Written Analogue Questionnaire sub-scale was regressed onto the group variable (DD vs. TD). These analyses were to establish whether there were group differences on any variable. MPlus employs Full Information Maximum Likelihood (FIML) to deal with missing data. In addition, maximum likelihood estimation with robust standard errors (MLR) provides estimates that are robust in the face of non-normality; this was used because of skew present on some variables. A more stringent significance level of *p* < .01 was used to adjust for the increased chance of Type I error when conducting a number of tests. The result of these analyses can be seen in Table 3.

**Table 3:** Group differences on the written analogue questionnaire

WAQ variable	Intercept (SE)	Unstandardized beta (SE)	Beta significance
Problem	5.20 (0.21)	−0.23 (0.35)	*p* = .511
Child control	7.52 (0.21)	−1.87 (0.35)	*p* < .001
Child responsibility	7.34 (0.21)	−2.51 (0.39)	*p* < .001
Blame	6.11 (0.27)	−2.12 (0.39)	*p* < .001
Intent	4.88 (0.23)	−1.18 (0.37)	*p* = .002
Parent control	5.31 (0.25)	0.51 (0.38)	*p* = .185
Parent responsibility	4.98 (0.24)	−0.78 (0.39)	*p* = .043
Perceived control	−2.30 (0.36)	2.47 (0.51)	*p* < .001
Anger	4.51 (0.26)	−0.88 (0.38)	*p* = .019

*Note*: The unstandardized beta reflects an estimate of the difference between the two groups; the group variable was coded TD = 0, DD = 1

The DD-group scored significantly lower on *child control*, *child responsibility*, *blame*, *intent*, and *perceived control* than the TD-group, with medium to large effect sizes. No significant differences between groups were found on *problem*, *parent control*, *parent responsibility*, and *anger*. As the DD and TD-groups differed in terms of *gender* and *income* (see Table 1), their effect on the dependent WAQ variables was tested. We repeated the preceding analyses, replacing group with gender, again setting significance at *p* < .01. *Gender* and *income* had no significant effect on any of the WAQ variables variables (all *p* ≥ .020).

To determine whether there was a curvilinear relation of child control with parenting, scatterplots were examined. The plots for both groups combined or separate did not show a curvilinear relation.

The direction of effects of relations between causal attributions and parenting strategies may be different between the DD and TD groups. Prior to evaluating the overall relation between causal attributions and parenting strategies, potential interactions between *group* and causal attributions on parenting strategies were therefore examined. If this indicated a potential interaction between group and a particular attribution on parenting strategy, then this interaction was added to the main model in the next stage. MPlus was again used to conduct a multiple regression for each WAQ item with *PS Total* as the dependent variable and the WAQ item, *group*, and the interaction term (WAQ item*group) as predictors. Applying the same principle as before, only those interactions significant at the *p* < .01 level were accepted as significant. One significant interaction between *group* and *child control* was found: B = 0.20 (SE = 0.06), *p* = 0.001, *R* ^*2*^ = .08. This interaction term was consequently included in the multiple regression models in the next section.

Next the overall relations between child-focused causal attributions and parenting strategies, and between parent-focused causal attributions and parenting strategies were evaluated through multiple regression analyses. SPSS was used to identify whether problems existed with multicollinearity (MPlus does not provide these). In the child-focused model, inspection of the eigenvalues only indicated multicollinearity between child control and the interaction between child control and group (Field 2005). Child control was therefore excluded, and the model was run again. Multicollinearity diagnostics were re-examined. The largest VIF was 2.97 and did not exceed 10, and the mean VIF was 2.06 and was not substantially greater than 1 (Bowerman and O’Connell 1990), which indicated that multicollinearity was not a problem for the final child-focused model. In the parent-focused model, the eigenvalues indicated multicollinearity between parent control and perceived control. Parent control was therefore excluded, and the model was run again. Multicollinearity diagnostics were re-examined. The largest VIF was 1.25 and did not exceed 10, the mean VIF was 1.15 and was not substantially greater than 1 (Bowerman and O’Connell 1990) which also indicated that multicollinearity was not a problem for the final parent-focused model. The final child- and parent-focused models were then run using MPlus and are presented in Table 4.

**Table 4:** Multiple regression of written analogue questionnaire variables on parenting scale

	Predictor	*B*	SE *B*	*β*	*p*-value
Child focused	Intercept	3.25	0.25		.000
	Group	−.08	0.04	−.06	.571
	Child responsibility	−0.16	0.04	−.56	.000
	Blame	0.09	0.03	.32	.003
	Intent	0.07	0.03	.22	.032
	Group*child control MC	0.12	0.05	.24	.019
Parent focused	Intercept	2.75	0.22		.000
	Group	−0.21	−0.16	−.13	.089
	Parent responsibility	−0.04	0.03	−.11	.221
	Perceived control	0.07	0.02	.33	.000
	Anger	0.12	0.03	.38	.000

*Note*: *Group* coded as TD = 0, DD = 1; *B* = beta; SE *B* = standard error of beta; *β* = standardized beta; MC = mean centered

**Fig 1:**
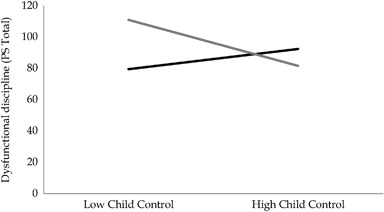
The relation between child control and discipline practices for the DD-group (*black* line) and the TD-group (*grey* line)

For the child-focused model, the predictor variables accounted for 16% of the variance (*R* ^2^ = .16) in the Parenting Scale. Child responsibility, blame, intent and the interaction term between child control and group were significant predictors of discipline strategies. Parents who viewed their child as more responsible, less to blame and as acting with less intent, reported using less dysfunctional discipline. Simple slopes analyses were carried out in SPSS to interpret the significant interaction (Aiken and West 1991). This revealed that the relation between *child control* and *PS Total* was positive but non-significant for the DD-group, *β* = .14, *t*(119) = 1.02, *p* = .31, and negative and significant for the TD-group, *β* = −.28, *t*(119) = −2.38, *p* < .05, (see Fig. 1). Parents in the TD-group who viewed their child as having more control, reported using less dysfunctional discipline, while a relation between child control and discipline practices was not found for the DD-group.

For the parent-focused model, the predictor variables accounted for 18% of the variance (*R* ^2^ = .18) in the Parenting Scale. Perceived control and anger were significant predictors of discipline practices. Parents who scored lower on *perceived control* and who felt less angry reported using less dysfunctional discipline.

## Discussion

Parents of children with DD viewed their child as having less control over, and less responsibility for, problematic behavior, and as acting with less intent and being less to blame than parents of TD children. This supports research showing that parents of children with DD view their child’s behavior relatively positively in terms of child responsibility and control (Chavira et al. 2000; Whittingham et al. 2008). It also suggests that these parents viewed their child’s behavior as an unavoidable part of DD (Armstrong and Dagnan 2011; Chavira et al. 2000; Whittingham et al. 2008). In contrast, both parents of children with DD and those with TD children attributed similar levels of control and responsibility for problematic behavior to themselves. While parents of children with DD seemed to excuse the child for problematic behavior, they continued to view themselves as responsible and in control.

Attributions of child control, responsibility, blame and intent were associated with parents’ report of their use of discipline practices. For both groups, blame and intent had a positive association with dysfunctional discipline practices, supporting Dix et al.’s (1986, 1989) and Baden and Howe’s (1992) findings. Attributions for child responsibility were, however, negatively related to dysfunctional discipline practices rather than positively as previously reported (Armstrong and Dagnan 2011; Chavira et al. 2000; Snarr et al. 2009), possibly because in previous studies responsibility was an aggregated construct comprised of constructs such as control, intent and blame. The current study demonstrated that responsibility and control are clearly distinct from each other and from blame and intent, with different relations with discipline practices. Future research should seek to ensure that these constructs are not conflated.

The relationship between child control and parents’ report of their use of discipline practices suggested that this differs by group. For the DD-group, no significant association was found between child control and discipline practices. In Whittingham et al.’s (2006) study, lower levels of control attributed to the child were related to parents finding behavior management strategies more usable, but seeing the value of management strategies is a different issue to the actual use of discipline practices. Possibly, the current study found no association for parents of children with DD because children with DD are generally perceived as having lower levels of control and therefore other attributions may be more strongly related to parents’ reactions to child behavior.

For the TD-group on the other hand, viewing the child as having more control was associated with the report of less dysfunctional discipline. This seems to be in contrast to past research that found a positive relationship between attributions of child control and negative parental emotions (Bugental et al. 1990; Dix et al. 1986; Johnston and Patenaude 1994). For the TD-group, seeing the child as having low control may be associated with viewing the child as less capable of learning positive behavior. This may reflect Woolfson’s (2005) parenting paradox, helping to explain more dysfunctional discipline.

Self-directed attributions were also associated with parent’s report of their use of discipline practices. *Perceived control* and *anger* related positively to dysfunctional discipline. Higher levels of perceived control, i.e. high parent control in combination with low child control, were associated with the use of more dysfunctional discipline. Bugental and colleagues have however repeatedly found mothers with low perceived control to be most at risk for coercive and harsh parenting (Bugental et al. 1989; Bugental and Happaney 2004). These studies though included mothers and their children who were at risk for child abuse. It may be that an imbalance between child and parent control in general is associated with dysfunctional practices, but that low perceived control is specifically of relevance to populations at risk for abuse, and high perceived control to other populations.

For the first time, our results allow identification of supportive parental attributions associated with lower levels of dysfunctional discipline practices for parents of children with a range of developmental delays. These include assigning low levels of blame and intent to the child, in combination with perceiving low levels of parent control. Such attributions enable parents to perceive their child and themselves in a positive light when problematic behavior takes place. Simultaneously, though, assigning high levels of responsibility to the child was also related to less dysfunctional discipline. This is more likely to reflect views of problematic behavior as not just a fixed part of the child’s DD, but in contrast, assigning some responsibility for the behavior to the child so that there is a possibility for change and improvement.

### Strengths and Limitations

Our focus was on attributions used by parents of children with developmental delays, and was informed by a social construction of developmental disability (e.g., Oliver 1996) rather than medical diagnostic categories. As a result, the findings apply to this wider group rather than only to specific diagnoses.

Participants’ children were aged 6 to 12 years. Younger children, of pre-school age, are seen as possessing less knowledge than school-aged children about what behavior is and is not appropriate, and so are viewed as having less control, and therefore parents do not become as upset with them for misbehaving as with older children (Dix et al. 1989; Johnston and Patenaude 1994). To assess any differences between parents of children with DD and parents of TD children, a sample aged six to twelve is more appropriate as parents of younger children in both groups are likely to share similar attributions that are related to age. As children enter puberty, parents expect to see many changes in their child’s behavior (Gretarsson and Gelfand 1988) and also have been found to hold different attributions, with their child’s behavior being seen as caused to a greater extend by influences of friends and school (Cote and Azar 1997).

Participation in the study was dependent on a convenience sample of volunteer parents. Using a convenience sample risks that the study sample may not be representative of the population from which it is drawn and that caution should be applied in generalizing the findings.

The ethnic composition of studies investigating causal attributions and parenting strategies among parents of children with DD has not always been described (e.g. Keenan et al. 2007; Whittingham et al. 2006, 2008). Participants taking part in Drysdale et al.’s (2009) and the current study were primarily white British while those in Chavira et al.’s study (2000) were all of Latin-American descent. Future research could address these issues by including information on the ethnic composition of their study sample, and by recruiting more ethnically diverse study samples that reflect the composition of the study population.

Vignettes used in this study were piloted, which demonstrated that parents could readily interpret the described situations and relate them to their own circumstances. This confirmed acceptable validity and reliability thus addressing problems that are sometimes raised about the ecological validity of vignette methodology (e.g., Armstrong and Dagnan 2011). In addition, both groups of parents were found to rate the behaviors described in the scenarios as similarly problematic, indicating that the interpretation of the behaviors was comparable between the two groups. Furthermore, using pre-specified vignettes meant that both groups of parents based their answers on the same behaviors and their responses could be directly compared. Social desirability may have been an issue for parents completing the PS, but Arnold et al. (1993) reported that mothers often indicated not knowing which alternative was the ‘correct’ response in this scale.

This study demonstrated that discipline practices used by parents of children with DD are associated with parent attributions of child responsibility, blame and intent, as well as parents’ perceived control for problematic child behavior. Attributions of child control may relate to discipline practices in distinctively different ways for parents of children with DD and TD children, although a study with a larger sample and more power needs to confirm these results.
